# A New Antiseptic Treatment of Wounds

**Published:** 1890-11-29

**Authors:** 


### SURGERY.

### A NEW ANTISEPTIC TREATMENT OF WOUNDS.

Dr. Oscar Block, senior surgeon to the Frederick Hospital, Copenhagen, has published in the last year, and the present, a paper upon antiseptics, and a second upon a new antiseptic treatment of wounds, which falls back almost indentically upon the strict Listerian method. The earlier monograph which appeared in the *Nordiskt Medicinsk Arkiv*, vol. 21, discussed the claims of the three antiseptics which are most in use—corrosive sublimate, iodoform, and carbolic acid, and after rejecting the former because of the risk of poisoning by intoxication, and because after all it is not quite effective in destroying the egg germs of the bacilli, and rejecting also iodoform, this distinguished authority accepted carbolic acid in a 3 per cent. solution as preferable to all other antiseptics. In the second monograph which is now before us, in a reprint from the *Nordiskt Medicinsk Arkiv*, for 1890, Dr. Block puts into practical application his views of the excellency of carbolic acid as an antiseptic, writes in praise of the old Listerian method, and does it the yet higher honour of accepting it almost in toto, exchanging only for the mackintosh covering, a covering of non-absorbent gauze. He addresses himself especially to practitioners who may have to deal with cases of operation at a distance from home in the country, for whom it is a desideratum that dressings shall be handy, portable, and effectual. Under these circumstances, then, he recommends that carbolic lotion of a strength represented by 3 per cent., or one part of acid to thirty-three of water be chosen as the antiseptic, and that gauze, with pads—inwardly next the skin absorbent, and externally non-absorbent, which have been previously sterilised by a method of his own—fixed with gauze bandages, also sterile, be chosen for the dressings. His gauze and wadding are absorbent and non-absorbent. Upon the skin, next the wound, absorbent, so that all discharges, and even small quantities of moisture are taken up, leaving the wound under what is practically a dry dressing; outside, enveloping the whole, non-absorbent, so that nothing noxious is admitted from the air. This covering acts as a protective, and replaces in this sense Professor Lister's mackintosh, to which it is superior, in the author's opinion, in effectiveness and convenience. And where does the carbolic acid come in? The absorbent gauze which is first applied upon the wound, forming its immediate covering is soaked in the 3 per cent. solution of carbolic acid, and wrung dry before application. The gauze to be sterilised—for it must be sterilised before packing in the surgeon's bag and being brought to the scene of operation—is taken in small quantities, made into a ball, and wrapped up in filtering-paper. The little bundle is then exposed to a current of steam, in a special stove constructed by Dr. Benzon, of Copenhagen, and patented by him, for a space of half-an-hour, under a high atmospheric pressure, and with a temperature of 120 deg. centigrade. The packets, when dry, are not unrolled, and the gauze continues to retain its property of sterility for any desired length of time. The dust collecting on the outside of the parcels does no harm, and may stay there until at the operation table the whole is unpacked by the side of the wound, stitched up, and prepared for the final dressing. Some absorbent gauze is then wrung out of the antiseptic carbolic acid solution, and applied next the skin, gentle elastic compression being made by absorbent gauze bandages. Then follow the layers of sterilised non-absorbent materials, carefully laid on and adjusted by outer bandages of non-absorbent dressing. This non-absorbent layer taken confidently from its sterilised packet in filtering-paper is innoxious as regards everything inside, and fully protective against influences from the atmosphere outside. The monograph runs to 65 pages in the original Danish, having, however, a French résumé attached, as, indeed, have all the papers in the *Northern Medical Journal*, and concludes with a narrative of a series of fifty cases treated by this method. Among these are examples of hernia and cancer operated upon, resections, and wounds of all kinds; and the results must be pronounced highly favourable. Categorically, the Copenhagen surgeon claims the following advantages for his procedure: The certain presence of the antiseptic, which is applied only in the presence of the operation, and, being in small quantity, avoids all risk of poisonous intoxication; secondly, that the dressing is a dry dressing, possessing recognised superiority as such; and, lastly, that it is inexpensive and conveniently portable for the medical man.

